# A cost-effectiveness analysis of the effect of hospital variation in the probability of providing treatment with curative intent in potentially curable esophageal and gastric cancer patients

**DOI:** 10.1093/dote/doaf057

**Published:** 2025-07-18

**Authors:** Saskia P M Truijen, Pauline A J Vissers, Grard A P Nieuwenhuijzen, Maurice J C van der Sangen, Peter D Siersema, Marije Slingerland, Nadia H Mohammad, Laurens V Beerepoot, Mark I van Berge Henegouwen, Pieter C van der Sluis, Camiel Rosman, Ewout A Kouwenhoven, Hüseyin Aktaş, Hanneke W M van Laarhoven, Carin A Uyl-de Groot, Rob H A Verhoeven

**Affiliations:** Department of Research and Development, Netherlands Comprehensive Cancer Organization (IKNL), Boven Clarenburg 2, 3511 CV, Utrecht, the Netherlands; Department of Rheumatology, Maastricht University Medical Center, and Care and Public Health Institute (CAPHRI), Maastricht University, P. Debyelaan 25, 6229 HX, Maastricht, the Netherlands; Department of Research and Development, Netherlands Comprehensive Cancer Organization (IKNL), Boven Clarenburg 2, 3511 CV, Utrecht, the Netherlands; Department of Surgery, Radboud University Medical Center, Geert Grooteplein Zuid 10, 6525 GA, Nijmegen, the Netherlands; Department of Surgery, Catharina Hospital, Michelangelolaan 2, 5623 EJ, Eindhoven, the Netherlands; Department of Radiation Oncology, Catharina Hospital, Michelangelolaan 2, 5623 EJ, Eindhoven, the Netherlands; Department of Gastroenterology and Hepatology, Radboud University Medical Center, Geert Grooteplein Zuid 10, 6525 GA, Nijmegen, the Netherlands; Department of Medical Oncology, Leiden University Medical Center, Albinusdreef 2, 2333 ZG, Leiden, the Netherlands; Department of Medical Oncology, University Medical Center Utrecht, Heidelberglaan 100, 3584 CX, Utrecht, the Netherlands; Department of Medical Oncology, Elisabeth-TweeSteden Hospital, Dr. Deelenlaan 5, 5042 AD, Tilburg, the Netherlands; Department of Surgery, Amsterdam University Medical Centers, location University of Amsterdam, De Boelelaan 1117, 1081 HV, Amsterdam, the Netherlands; Cancer Center Amsterdam, Cancer Treatment and Quality of Life Amsterdam, De Boelelaan 1118, 1081 HV, Amsterdam, the Netherlands; Department of Surgery, Erasmus Medical Center, Dr. Molewaterplein 40, 3015 GD, Rotterdam, the Netherlands; Department of Surgery, Radboud University Medical Center, Geert Grooteplein Zuid 10, 6525 GA, Nijmegen, the Netherlands; Department of Surgery, Ziekenhuisgroep (Hospital Group) Twente, Zilvermeeuw 1, 7609 PP, Almelo, the Netherlands; Department of Gastroenterology and Hepatology, Ziekenhuisgroep (Hospital Group) Twente, Zilvermeeuw 1, 7609 PP, Almelo, the Netherlands; Cancer Center Amsterdam, Cancer Treatment and Quality of Life Amsterdam, De Boelelaan 1118, 1081 HV, Amsterdam, the Netherlands; Department of Medical Oncology, Amsterdam University Medical Center location University of Amsterdam, De Boelelaan 1117, 1081 HV, Amsterdam, the Netherlands; Erasmus School of Health Policy and Management, Erasmus University Rotterdam, Burgemeester Oudlaan 50, 3062 PA, Rotterdam, the Netherlands; Institute for Medical Technology Assessment, Erasmus University Rotterdam, Burgemeester Oudlaan 50, 3062 PA, Rotterdam, the Netherlands; Department of Research and Development, Netherlands Comprehensive Cancer Organization (IKNL), Boven Clarenburg 2, 3511 CV, Utrecht, the Netherlands; Cancer Center Amsterdam, Cancer Treatment and Quality of Life Amsterdam, De Boelelaan 1118, 1081 HV, Amsterdam, the Netherlands; Department of Medical Oncology, Amsterdam University Medical Center location University of Amsterdam, De Boelelaan 1117, 1081 HV, Amsterdam, the Netherlands

**Keywords:** cost-effectiveness analysis, esophageal neoplasms, stomach neoplasms, hospitals, probability

## Abstract

For potentially curable esophageal cancer (EC) and gastric cancer (GC) patients, the probability of treatment with curative intent varies between hospitals and is associated with survival. This study examines the effect of this variation on health economics outcomes and cost-effectiveness. We performed a cost-effectiveness analysis from a societal perspective in potentially curable EC or GC patients selected from the Netherlands Cancer Registry. Resource use and costs were estimated for each treatment strategy from diagnosis until five years follow-up using a top–down costing method. Hospitals were divided into tertiles of low, medium, or high probability of treatment with curative intent using multilevel multivariable logistic regression. The primary outcome was the incremental cost-effectiveness ratio (ICER). Mean total costs per patient was not significantly different between low, medium, and high probability hospitals for EC (*n* = 9468) (€47,532 vs. €47,384 vs. €47,825), while for GC (*n* = 3085) costs were significantly lower in low compared to medium and high probability hospitals (€27,759 vs. €30,183 vs. €29,589, both *P* < 0.001). Costs per quality adjusted life year (QALY) were slightly lower in high probability hospitals for both EC and GC (EC: €29,181 vs. €28,646 vs. €27,659, GC: €25,003 vs. €22,505 vs. €20,495). ICERs were highest for high vs. medium probability hospitals for EC (€4900/QALY) and for medium vs. low probability hospitals for GC (€10,539/QALY). Variation in treatment with curative intent between hospitals affects health economics outcomes to a limited extent. Although all hospital comparisons were cost-effective, for the highest QALY gain, it is recommended to treat potentially curable patients as in high probability hospitals.

## INTRODUCTION

Esophageal cancer (EC) and gastric cancer (GC) were the 7th and 5th most frequently diagnosed cancers worldwide in 2020, leading to 769,000 and 544,000 deaths globally.^1^ Both types of cancer are often diagnosed when they have already metastasized, leading to poor 5-year survival rates of approximately 23% for GC and EC.^2^^,^^3^ Only 62% of patients with EC and 56% of patients with GC are potentially curable at diagnosis.^4^ At this moment, the cornerstone of curative treatment is surgery with or without (neo)adjuvant chemo(radio)therapy, definitive chemoradiation for patients with EC unfit for surgery or endoscopic resection for patients with early cancers.

For potentially curable patients (cT1-4A, X, any cN, cM0), the probability of being treated with curative intent varies largely between hospitals in the Netherlands,^4–6^ with proportions ranging from 54%–89% for EC and 45%–100% for GC in the period 2015–2017.^4^ Previous studies showed that this variation is associated with survival, as patients diagnosed in a hospital with a high probability of being treated with curative intent have a higher survival compared to patients diagnosed in a hospital with a low probability of being treated with curative intent.^4–6^ Since the costs involved in curative and non-curative treatments probably differ, this variation might also have consequences for the costs of cancer care, which are still unknown.

A study in the US showed that the costs of cancer care are particularly high in the first year after diagnosis, with EC and GC belonging to the cancer types with the highest first-year costs.^7^ It is hypothesized that the aforementioned variation affects both direct and indirect costs (i.e. indirect medical costs, informal care costs, productivity loss costs). For patients with EC surviving at least one year after diagnosis, costs were more than twice as high compared to those dying within the first year.^8^ Hospitals treating a high proportion of patients with curative intent are thus expected to have more costs than those treating a lower proportion of patients with curative intent. The exact association between variation and costs, and whether the difference in costs between hospitals is relevant, has as far as we know not been studied previously.

Therefore, this study aims to perform a cost-effectiveness analysis to compare the costs per quality adjusted life year (QALY) between hospitals. More specifically, we intended to determine the effect of variation in treatment with curative intent between hospitals in patients with potentially curable EC or GC on health economics outcomes.

## METHODS

### Study design and population

An observational top down costing study was conducted from a societal perspective for the period from diagnosis until five years thereafter, according to the methodology described in the Dutch Manual for Costing in Economic Evaluations (2015).^9^ Patient data were obtained from the Netherlands Cancer Registry (NCR), a nationwide population-based cancer registry including all cancer patients in the Netherlands, in which trained data-managers extract information from medical records. Data from the Prospective Observational Cohort Study of Esophageal-Gastric Cancer Patients (POCOP) study,^10^ in which patients with EC and GC are prospectively included and receive questionnaires on patient reported outcomes at regular intervals, were linked to the NCR. Patients with potentially curable EC (C15.x), including gastro-esophageal junction/cardia (C16.0),^11^ and GC (C16.1–16.9^11^) diagnosed between January 2015 until June 2020 were included. Potentially curable disease was defined as stage cT1-4A, X, any cN, cM0, according to the Union for International Cancer Control (UICC) TNM classification of malignant tumors, 7^th^ (yr. 2015–2016) and 8^th^ edition (yr. 2017–2020).^12^^,^^13^ Exclusion criteria were diagnosis in a hospital with <10 diagnoses during the study period (*n* = 43), patients undergoing chemoradiation with an unknown radiation dose (*n* = 228), no data on overall survival (*n* = 16*)*, and patients who died on the diagnosis date (*n* = 18). Comorbidities were categorized as 0, 1, or ≥ 2 comorbidities according to the modified Charlson Comorbidity Index (CCI).^14^ Performance status was measured by the Eastern Cooperative Oncology Group (ECOG) scale^15^ and categorized as 0, 1, 2, or 3/4. This study was approved by the Privacy Review Board of the NCR and the scientific committee of the Dutch Upper GI Cancer Group. No ethical approval was required according to the Central Committee on Research involving Human Subjects.

In the Netherlands, all hospitals providing EC and GC care operate within a universal, publicly accessible healthcare system, without private EC and GC care. Diagnosis and treatment is therefore not dependent on patient’s income or insurance. Furthermore, curative surgery is centralized to hospitals performing ≥20 resections per year (EC: *n* = 21, GC: *n* = 16).

### Outcome measures

The primary outcome measure was the incremental cost-effectiveness ratio (ICER) (∆costs/∆QALY), which was compared between hospitals with a low, medium, or high probability of treatment with curative intent (more details in section 2.5), from both a societal and healthcare perspective. Total costs comprised direct and indirect costs; direct costs included medical costs, and indirect costs included travel expenses and costs of productivity loss. QALY is frequently used in cost-effectiveness analyses in which a life year is corrected with a factor for quality of life. A value of 1 represents ‘perfect health’ whereas a value of 0 represents ‘death’.^16^ Disease burden was calculated by the iMTA Disease Burden Calculator^17^ and ranged between 0.71–1.00. A threshold of €80,000/QALY^18^ was used to determine whether the strategy is cost-effective.

### Treatment groups

Treatment groups for analyses included for EC (1) neoadjuvant chemoradiotherapy (nCRT) with resection, (2) resection (with or without (neo)adjuvant treatment other than nCRT), (3) endoscopic resection, (4) definitive chemoradiation (dCRT) (radiotherapy dose ≥42 Gy), (5) nCRT without resection (radiotherapy dose 41.4 Gy), (6) radiotherapy only, (7) systemic therapy only, and (8) best supportive care (incl. stents), and for GC (1) neoadjuvant (NACT) and adjuvant chemotherapy (ACT) with resection, (2) NACT with resection, (3) resection (without peri-operative treatment), (4) endoscopic resection, (5) systemic therapy only, and (6) best supportive care (incl. stents). EC group five consists of patients in whom surgery was cancelled after nCRT because of the patient’s condition, progression of disease, or clinical complete response after nCRT. Treatment initiated with curative intent included groups 1–5 for both EC and GC, excluding mono- or doublet chemotherapy regimens in GC group 5. In this group, triplet chemotherapy regimens were considered as treatment initiated with curative intent as the included regimens are in general only given to patients with a good condition, making it very likely that this was intended as neoadjuvant treatment.

### Data collection

#### Quality adjusted life year

Mean number of QALYs was estimated for each treatment category. Mean utility values for each treatment category and time period were measured in patients included in the POCOP study (EC: *n* = 1529, GC: *n* = 229) with the EuroQoL five-dimension, five-level health status questionnaire (EQ-5D-5L).^19^ The five dimensions are mobility, self-care, usual activities, pain/discomfort, and anxiety/depression, with each consisting of five levels (no, slight, moderate, severe, or extreme problems). The Dutch standard set was applied to calculate the utilities for each time period.^20^ The following time periods were selected: (1) diagnosis - start primary treatment, (2) start - end primary treatment, (3) end primary treatment - six months after the end of primary treatment, (4) six - twelve months after the end of primary treatment, (5) ≥12 months after the end of primary treatment. In case of low numbers of available utility values, mean utilities of the most comparable treatment group were used. For period five, the number of life years was calculated as the mean time spent in period five of patients diagnosed in 2015–2016, who had the longest follow-up (FU) time.

#### Resource use

For the diagnostic and treatment period, the use of healthcare resources was estimated by exploring healthcare pathways for EC and GC patients of three hospitals, which was congregated into one pathway after consultation of medical specialists. NCR data was used for details about the received treatment and mean hospital days after resection. Units and proportions of patients undergoing additional diagnostics, medical consultations, and staging scans were estimated with NCR data of a subpopulation of patients participating in the VARIATE study (EC: *n* = 1492, GC: *n* = 1663), in which additional data was registered for the diagnostic period.^21^ Data on the proportion of patients admitted to the hospital after toxicity due to chemo- or radiotherapy were measured in an NCR subpopulation (EC: *n* = 132, GC: *n* = 45), and the mean number of hospital days were estimated in consultation with medical specialists.

The use of healthcare resources during the FU period was estimated by consulting the Dutch Guideline Database.^22^^,^^23^ For patients entering the palliative phase, the units of medical consultations, diagnostics, medication, and hospital stay were estimated from literature.^24–26^ For each treatment category, the proportion of patients developing local or distant recurrences after primary treatment, possible secondary treatment, and the mean number of months in FU and/or palliative phase were estimated with NCR subpopulation data of patients diagnosed in 2015 and 2016 (EC: *n* = 2419, GC: *n* = 807), for whom additional FU data was collected in the 2nd half of 2019.^27^ For patients undergoing only systemic therapy, FU data was also available for patients diagnosed in 2017. No additional FU data was available for patients receiving best supportive care or only radiotherapy; however, in case of recurrence often no additional treatment is given.^27^

#### Cost prices

Cost prices were obtained from the Dutch Manual for Costing: Methods and Standard Costs for in Economic Evaluations in Health Care (2015),^9^ the Dutch Healthcare Authority (NZa),^28^ hospital price lists^29^^,^^30^ and literature.^25^^,^^31–36^ Detailed references of costs are shown in Supplementary Table 1. Costs were estimated from diagnosis until death or end of FU to a maximum of five years after diagnosis. Costs were adjusted to 2020 using the Consumer Price Index (CPI).^37^

Direct medical costs included all medical hospital costs for EC/GC from diagnosis to end of FU, including costs of diagnostics, medical consultations, treatment, complications, hospital stay, palliative care and treatment after recurrence or progression. The calculation of travel costs is explained in Supplementary Table 1. Costs of productivity loss and replacement due to illness, disability or death were calculated using the friction cost approach with a friction period of 12 weeks.^9^ The Work Productivity and Activity Impairment—General Health (WPAI-GH) questionnaire^38^ was used to estimate absenteeism (=work time missed), presenteeism (=health-related impairment while working), and the proportion of patients performing paid work per treatment group in patients included in the POCOP study (EC: *n* = 1532, GC: *n* = 231). Costs of productivity loss were calculated by adding up costs of absenteeism and presenteeism, while considering the proportion of patients per treatment group performing paid work (Supplementary Table 2).

### Statistical analyses

Hospitals of diagnosis were divided into the tertiles low, medium or high probability of providing treatment with curative intent, according to the hospital’s adjusted odds ratio (OR) of providing treatment with curative intent, separately for EC and GC. Multilevel logistic regression analyses with random intercepts were used to analyze the hierarchically structured data. The dependent variable was being treated with curative intent or not. The covariates sex, age category, tumor histology, cT and cN stage, performance status, CCI score, and BMI were included to adjust for case-mix differences. A total of 81 and 75 hospitals were included for EC and GC, respectively.

Descriptive statistics of patient- and tumor characteristics were calculated, stratified by the hospital categories (low, medium, high), and expressed as frequencies and percentages (%). Differences between hospital categories were tested using chi-square tests.

A cost-effectiveness analysis (CEA) was performed. Total societal costs were calculated for each treatment, by summing the direct medical costs, travel costs, and costs of productivity loss. The proportion of patients using a particular resource within the treatment groups was taken into account. Each patient in the same treatment group received the same cost price. Mean costs per patient per hospital category were calculated as the times treatments were given multiplied by the treatment cost and divided by the total number of patients in the particular hospital category. Costs per QALY were calculated by dividing the total costs by the mean QALY of each treatment group. Differences in costs between the three hospital categories were tested using non-parametric Kruskal–Wallis tests.

The proportion of patients surviving the first year after diagnosis to provide context to treatment costs and costs per QALY were derived using Kaplan–Meier curves. Sensitivity analyses were conducted on the parameters with possible major impact on the results: a −15% and +15% change in either resection price, hospital day price, palliative phase (€/month) price, or QALY.

Statistical significance was based on two-sides values of *P* < 0.05, unless otherwise stated. Statistical analyses were performed by using SAS® version 9.4 (SAS Institute Inc., Cary, NC, USA).

**Table 1:** Characteristics of EC and GC patients according to hospital adjusted probability of treatment with curative intent

	Esophageal cancer					Gastric cancer				
	Hospital adjusted probability of treatment with curative intent					Hospital adjusted probability of treatment with curative intent				
	Total	Low	Medium	High	*P*-value	Total	Low	Medium	High	*P*-value
Number of patients	9468 (100.0)	3349 (100.0)	2851 (100.0)	3268 (100.0)	<0.001	3085 (100.0)	904 (100.0)	1163 (100.0)	1018 (100.0)	<0.001
Sex, male	6862 (72.5)	2406 (71.8)	2076 (72.8)	2380 (72.8)	0.59	1835 (59.5)	526 (58.2)	682 (58.6)	627 (61.6)	0.24
Age (year)					0.63					0.09
<60	1453 (15.4)	524 (15.7)	429 (15.1)	500 (15.3)		427 (13.8)	109 (12.1)	181 (15.6)	137 (13.5)	
60–74	4828 (51.0)	1683 (50.3)	1447 (50.8)	1698 (52.0)		1093 (35.4)	310 (34.3)	405 (34.8)	378 (37.1)	
≥75	3187 (33.7)	1142 (34.1)	975 (34.2)	1070 (32.7)		1565 (50.7)	485 (53.7)	577 (49.6)	503 (49.4)	
cT Classification					<0.001					0.22
cT1	483 (5.1)	160 (4.8)	130 (4.6)	193 (5.9)		134 (4.3)	30 (3.3)	51 (4.4)	53 (5.2)	
cT2	2400 (25.4)	849 (25.4)	668 (23.4)	883 (27.0)		941 (30.5)	271 (30.0)	379 (32.6)	291 (28.6)	
cT3	4909 (51.9)	1804 (53.9)	1538 (54.0)	1567 (48.0)		927 (30.1)	287 (31.8)	337 (29.0)	303 (29.8)	
cT4A^a^	181 (1.9)	57 (1.7)	54 (1.9)	70 (2.1)		187 (6.1)	50 (5.5)	66 (5.7)	71 (7.0)	
cTX	1495 (15.8)	479 (14.3)	461 (16.2)	555 (17.0)		896 (29.0)	266 (29.4)	330 (28.4)	300 (29.5)	
cN Classification					0.004					0.28
cN0	3970 (41.9)	1342 (40.1)	1183 (41.5)	1445 (44.2)		1795 (58.2)	534 (59.1)	684 (58.8)	577 (56.7)	
cN+	4635 (49.0)	1715 (51.2)	1393 (48.9)	1527 (46.7)		913 (29.6)	254 (28.1)	354 (30.4)	305 (30.0)	
cNX	863 (9.1)	292 (8.7)	275 (9.7)	296 (9.1)		377 (12.2)	116 (12.8)	125 (10.8)	136 (13.7)	
No. of comorbidities					<0.001					0.003
0	3695 (39.0)	1364 (40.7)	1127 (39.5)	1204 (36.8)		1124 (36.4)	333 (36.8)	393 (33.8)	398 (39.1)	
1	3007 (31.8)	1071 (32.0)	912 (32.0)	1024 (31.3)		991 (32.1)	289 (32.0)	369 (31.7)	333 (32.7)	
≥3	2226 (23.5)	781 (23.3)	713 (25.0)	732 (22.4)		748 (24.3)	234 (25.9)	296 (25.5)	218 (21.4)	
Unknown	540 (5.7)	133 (4.0)	99 (3.5)	308 (9.4)		222 (7.2)	48 (5.3)	105 (9.0)	69 (6.8)	
Performance status (ECOG)					<0.001					<0.001
0	3067 (32.4)	1120 (33.4)	1049 (36.8)	898 (27.5)		795 (25.8)	247 (27.3)	302 (26.0)	246 (24.2)	
1	2825 (29.8)	1085 (32.4)	824 (28.9)	916 (28.0)		768 (24.9)	256 (28.3)	273 (23.5)	239 (23.5)	
2	827 (8.7)	328 (9.8)	240 (8.4)	259 (7.9)		225 (7.3)	62 (6.9)	91 (7.8)	72 (7.1)	
3 or 4	357 (3.8)	111 (3.3)	121 (4.2)	125 (3.8)		140 (4.5)	51 (5.6)	58 (5.0)	31 (3.1)	
Unknown	2392 (25.3)	705 (21.1)	617 (21.6)	1070 (32.7)		1157 (37.5)	288 (31.9)	439 (37.8)	430 (42.2)	
Diagnosed in expert center^b^, yes	2898 (30.6)	946 (28.3)	381 (13.4)	1571 (48.1)	<0.001	921 (29.9)	179 (19.8)	395 (34.0)	347 (34.1)	<0.001
Treatment with curative intent, yes	6877 (72.6)	2352 (70.2)	2076 (72.8)	2449 (74.9)	<0.001	2129 (69.0)	553 (61.2)	809 (69.6)	767 (75.3)	<0.001
Type of treatment received					<0.001					<0.001
nCRT and resection	3506 (37.0)	1247 (37.2)	1042 (36.6)	1217 (37.2)		–	–	–	–	
NACT with resection	–	–	–	–		498 (16.1)	150 (16.6)	191 (16.4)	157 (15.4)	
NACT+ACT with resection	–	–	–	–		635 (20.6)	156 (17.3)	278 (23.9)	201 (19.7)	
Resection, other^c^	779 (8.2)	265 (7.9)	207 (7.3)	307 (9.4)		783 (25.4)	178 (19.7)	270 (23.2)	335 (32.9)	
Endoscopic resection	600 (6.3)	171 (5.1)	170 (6.0)	259 (7.9)		92 (3.0)	26 (2.9)	26 (2.2)	40 (3.9)	
dCRT	1252 (13.2)	435 (13.0)	401 (14.1)	416 (12.7)		–	–	–	–	
nCRT without resection	740 (7.8)	234 (7.0)	256 (9.0)	250 (7.7)		–	–	–	–	
Radiotherapy only	1130 (11.9)	454 (13.6)	326 (11.4)	350 (10.7)		–	–	–	–	
Systemic therapy only	135 (1.4)	47 (1.4)	39 (1.4)	49 (1.5)		173 (5.6)	59 (6.5)	67 (5.8)	47 (4.6)	
Best supportive care	1326 (14.0)	496 (14.8)	410 (14.4)	420 (12.9)		904 (29.3)	335 (37.1)	331 (28.5)	238 (23.4)	

^a^Includes patients staged as cT4B after explorative surgery.

^b^Expert centers are hospitals performing rminEC and/or GC surgeries per year.

^c^For patients with EC, this group includes patients undergoing only resection, NACT with resection, or NACT+ACT with resection. For patients with GC, this group includes solely patients undergoing only resection.

ECOG: Eastern Cooperative Oncology Group; nCRT: Neoadjuvant chemoradiation; NACT: Neoadjuvant chemotherapy; ACT: Adjuvant chemotherapy; dCRT Definitive chemoradiation.

## RESULTS

### Patient characteristics

The study population consisted of 9468 EC and 3085 GC patients (Table 1). Patients with EC in high probability hospitals more often had cT1 or cT2 and cN0 stage tumor compared to the other probability categories (cT: *P* < 0.001, cN: *P* = 0.004). The number of comorbidities and performance status differed between the hospital categories for both EC and GC (EC: both *P* < 0.001, GC: *P* = 0.003 and *P* < 0.001, respectively). In the low, medium, and high probability hospital categories a total of 70.2%, 72.8%, and 74.9% EC, and 61.2%, 69.6%, and 75.3% GC patients were treated with curative intent, respectively.

### Costs per treatment group

For EC, total and direct medical costs were highest for patients undergoing nCRT with resection (€75,559 and €63,116, respectively) (Table 2). For GC, total costs were highest for patients undergoing NACT+ACT with resection (€46,440), while direct medical costs were highest for NACT with resection (€36,850). For both EC and GC, lowest total costs were observed for patients undergoing endoscopic resection (€11,414 and €8964, respectively), followed by those receiving best supportive care (€15,079 and €15,523, respectively). Travel costs were highest for EC in the treatment groups involving chemoradiation (€1460), whilst for GC travel costs were highest for patients with NACT+ACT with resection (€838). Travel costs were lowest for those receiving best supportive care (EC: €337, GC: €330). Highest costs of productivity loss were found in the EC resection (other) group (€12,610) and the GC NACT+ACT with resection group (€10,223).

**Table 2:** Total costs (2020 €) per EC or GC patient for each treatment category

Treatment	Number of patients	Alive 1 year after diagnosis (%)	Recurrence or progression (%)^a^	€/QALY	Total costs	Direct medical costs	Travel costs	Productivity loss costs
Esophageal cancer								
nCRT and resection	3506	87.1	44.2	€26,326	€75,559	€63,116	€1460	€10,983
Resection, other	779	85.9	40.2	€19,896	€56,390	€42,923	€857	€12,610
Endoscopic resection	600	96.1	13.5	€3516	€11,414	€10,731	€683	–^b^
Definitive chemoradiation	1252	74.5	53.5	€19,059	€37,692	€31,971	€1084	€4637
nCRT without resection	740	63.3	69.1	€28,021	€45,094	€36,553	€1238	€7303
Radiotherapy only	1130	36.9	NA	€36,823	€27,825	€26,888	€688	€249
Systemic therapy only	135	37.5	53.5	€27,853	€20,755	€19,411	€475	€869^c^
Best supportive care	1326	15.0	NA	€52,723	€15,079	€14,742	€337	€0^d^
Gastric cancer								
NACT+ACT with resection	635	93.4	40.3	€14,032	€46,440	€35,379	€838	€10,223
NACT with resection	498	82.2	42.8	€15,939	€42,336	€36,850	€677	€4809
Resection, other	783	76.3	36.4	€12,010	€27,205	€26,651	€554	–^b^
Endoscopic resection	92	94.5	7.4	€2923	€8964	€8241	€723	–^b^
Systemic therapy only	173	29.0	43.4	€33,308	€20,738	€16,322	€375	€4041^c^
Best supportive care	904	21.4	NA	€41,324	€15,523	€15,193	€330	€0^d^

^a^Recurrence or progression within five years after diagnosis.

^b^No data were available.

^c^Consists only productivity loss costs of period 1 for EC, and costs of period 1, 2 and 3 for GC, because of the unavailability of data.

^d^No data were available, it was assumed no productivity loss costs were made after considering age and survival probability.

nCRT: neoadjuvant chemoradiation; NACT: neoadjuvant chemotherapy; ACT: adjuvant chemotherapy.

Mean QALYs per treatment group are shown in Supplementary Table 3. When comparing total costs per QALY (Table 2), costs were highest in the best supportive care groups (EC: €52,723/QALY, GC: €41,324/QALY) and lowest for those undergoing endoscopic resection (EC: €3516/QALY, GC: €2923/QALY for both types of cancer.

### Costs according to hospital categories

Table 3 shows the mean costs per patient and the mean QALY, stratified by hospitals with a low, medium, and high probability of providing treatment with curative intent. Mean total costs per patient were similar between patients diagnosed in low, medium, and high probability hospitals for EC (€47,532 vs. €47,384 vs. €47,825) and significantly higher for patients diagnosed in medium and high probability hospitals for GC (€27,759 vs. €30,183 vs. €29,589, *P* < 0.001). Mean total costs per QALY per patient were significantly lower for patients diagnosed in high probability hospitals compared to low and medium probability hospitals (EC: €29,181 vs. €28,646 vs. €27,659, high vs. low: *P* < 0.001 and high vs. medium: *P* = 0.008, GC: €25,003 vs. €22,505 vs. €20,495, both *P* < 0.001).

**Table 3:** Mean costs (€) per patient, stratified by hospital adjusted probability of treatment with curative intent^a^

Cost categories	Hospital adjusted probability of treatment with curative intent					
	Low (1)	Medium (2)	High (3)	*p*-value		
Esophageal cancer				1 vs. 2	1 vs. 3	2 vs. 3
Direct medical costs, total	€40,263 (19,393)	€40,072 (19,345)	€40,326 (19,580)	0.99	0.97	0.91
Diagnosis	€2444 (0)	€2444 (0)	€2444 (0)	1.00	1.00	1.00
Treatment	€26,720 (20,790)	€26,590 (20,578)	€27,163 (20,603)	0.98	0.69	0.55
Follow-up	€438 (577)	€461 (611)	€523 (682)	0.75	<0.001^*^	<0.001^*^
Palliative care	€10,661 (3742)	€10,577 (3755)	€10,197 (3854)	0.91	<0.001^*^	<0.001^*^
Travel costs	€1023 (415)	€1034 (410)	€1035 (405)	0.82	0.80	1.00
Productivity loss costs	€6246 (5003)	€6278 (4905)	€6463 (4993)	0.96	0.30	0.19
Total costs	€47,532 (24,429)	€47,384 (24,295)	€47,825 (24,157)	0.99	0.97	0.91
Mean QALY	1.98 (1.05)	2.01 (1.03)	2.10 (1.02)	0.71	<0.001^*^	0.003^*^
€/QALY	€29,181 (12,123)	€28,646 (12,215)	€27,659 (12,317)	0.32	<0.001^*^	0.008^*^
Direct medical €/QALY	€26,066 (12,809)	€25,485 (12,800)	€24,496 (12,748)	0.43	<0.001^*^	0.001^*^
Gastric cancer				1 vs. 2	1 vs. 3	2 vs. 3
Direct medical costs, total	€24,400 (9529)	€26,145 (9284)	€26,068 (8931)	<0.001^*^	0.001^*^	0.93
Diagnosis	€1807 (600)	€1889 (622)	€1824 (605)	0.007^*^	0.82	0.04^*^
Treatment	€12,855 (11,146)	€15,042 (10,723)	€15,651 (10,101)	<0.001^*^	<0.001^*^	0.99
Follow-up	€360 (465)	€401 (418)	€463 (493)	<0.001^*^	<0.001^*^	0.07
Palliative care	€9378 (3563)	€8813 (3353)	€8131 (3448)	<0.001^*^	<0.001^*^	<0.001^*^
Travel costs	€534 (194)	€572 (196)	€575 (179)	<0.001^*^	<0.001^*^	0.964
Productivity loss costs	€2826 (3869)	€3466 (4210)	€2947 (4036)	0.003^*^	0.94	0.009^*^
Total costs	€27,759 (12,829)	€30,183 (12,884)	€29,589 (12,236)	<0.001^*^	<0.001^*^	0.74
Mean QALY	1.73 (1.20)	1.96 (1.18)	2.05 (1.08)	<0.001^*^	<0.001^*^	0.96
€/QALY	€25,003 (13,605)	€22,505 (12,886)	€20,495 (12,507)	<0.001^*^	<0.001^*^	<0.001^*^
Direct medical €/QALY	€23,240 (13,795)	€20,647 (13,048)	€18,894 (12,467)	<0.001^*^	<0.001^*^	0.07

^a^Costs 2020€ are presented as mean (SD).

^*^Statistically significant.

When considering separate cost categories, direct medical, travel, and productivity loss costs did not differ significantly between hospital categories for EC (€40,263 vs. €40,072 vs. €40,326, €1023 vs. €1034 vs. €1035, €6246 vs. €6278 vs. €6463, respectively, all *P* > 0.05). For GC, direct medical and travel costs were significantly lower for patients diagnosed in low probability hospitals compared to medium and high probability hospitals (€24,400 vs. €26,145 vs. €26,068 and €534 vs. €572 vs. €575, respectively, all *P* < 0.001). Productivity loss costs were significantly higher for patients diagnosed in medium compared to low and high probability hospitals (€2826 vs. €3466 vs. €2947, *P* = 0.003 and *P* = 0.009).

### ICER according to hospital categories

For EC, comparing the high vs. medium probability hospitals the ICERs amounted to €4900 per QALY gained for the societal perspective and €2822 per QALY gained for the healthcare perspective (Table 4). The ICERs of medium vs. low probability hospitals and high vs. low probability hospitals were lower for both perspectives. For GC, highest ICERs were found in medium vs. low probability hospitals (societal: €10,539/QALY and healthcare: €7587). In this group comparing high vs. medium probability hospitals led to cost savings and gain in QALYs for both perspectives. All ICERs were far below the threshold of €80,000/QALY for cost-effectiveness.

**Table 4:** ICERs from a societal and healthcare perspective per hospital adjusted probability of treatment with curative intent

Perspective	Hospital adjusted probability of treatment with curative intent		
	Medium (2) vs. low (1)	High (3) vs. low (1)	High (3) vs. medium (2)
Esophageal cancer			
ICER (total €/QALY)	–€4933	€2442	€4900
ICER (direct medical €/QALY)	–€6367	€525	€2822
Gastric cancer			
ICER (total €/QALY)	€10,539	€5719	–€6600
ICER (direct medical €/QALY)	€7587	€5213	–€856

### Sensitivity analyses

Sensitivity analyses showed that all ICERs remained far below the threshold for cost-effectiveness of €80,000/QALY despite a −15% and +15% change in either resection price, hospital day price, palliative phase (€/month) price, or QALY (Supplementary Table 4).

## DISCUSSION

This study showed that variation in the probability of providing treatment with curative intent between hospitals affects costs for potentially curable EC and GC only to a limited extent. The mean total costs per patient, including direct medical-, travel-, and productivity-loss costs, did not differ significantly between patients diagnosed in low, medium, and high probability hospitals for EC. Total costs for GC were significantly lower for patients diagnosed in low probability hospitals compared to medium and high probability hospitals. However, costs per QALY were slightly higher for patients diagnosed in low compared to high probability hospitals. Comparable results were observed when considering a healthcare perspective. Regardless of a number of significant comparisons, costs differed only to a limited extent between the three hospital categories. From both a societal and healthcare perspective all strategies can be considered as cost-effective, although treatment in high probability hospitals is favorable due to the higher QALY.

To our knowledge, this is the first study examining the effect of treatment variation between hospitals on health economics outcomes in patients with EC or GC. Moreover, European studies on costs regarding EC and GC are scarce, especially those with a societal perspective. Our study showed that direct medical costs for EC ranged from €10,731 to €63,116, and for GC from €8241 to €36,850, depending on the type of treatment. In a US study, costs for nCRT and resection were almost comparable to chemoradiotherapy alone ($47,389 vs. $46,659),^39^ whereas in our study costs for nCRT and resection were twice as high as for definitive chemoradiation (€63,116 vs. €31,971). This difference might be explained by a different time frame (diagnosis to 90 days after completion of therapy in the US study vs. diagnosis until death or end of FU in our study) and different chemotherapy regimens used. Costs for hospital days for definitive chemoradiation in our study might have been underestimated, as we had no treatment-specific data on hospital days after chemo- and/or radiotherapy toxicity. In the US study patients receiving definitive chemoradiation required more hospital days than patients receiving resection.^39^

Except for endoscopic resections, treatments with curative intent had as expected higher costs than palliative treatments, mainly caused by surgery and its accompanying hospital stay. In addition, a substantial proportion of patients undergoing treatment with curative intent have progression or recurrence after a certain period (Table 2), leading to costs for possible secondary treatment and the palliative phase. However, palliative care costs were still slightly higher for patients diagnosed in low probability hospitals, in which more patients were treated without curative intent and thus received palliative care immediately after diagnosis, in comparison with patients diagnosed in high probability hospitals (EC: €10,661 vs. €10,197, GC: €9378 vs. €8131). In patients with GC, direct medical costs of treatment with NACT with resection (€36,850) were higher than those for NACT+ACT with resection (€35,379), mainly caused by a higher number of hospital days after resection in the NACT with resection group (14.9 vs. 8.5 days). Costs of productivity loss were highest for treatments involving resection for EC and NACT+ACT with resection for GC, as in these treatment groups patients are generally younger and often in a better condition, and thus performing more often paid work compared to patients undergoing palliative treatments. Moreover, these treatment groups involve longer treatment periods and thus a higher risk of productivity loss. Higher productivity loss costs were observed for patients diagnosed in medium compared to low and high probability hospitals for GC, probably explained by a higher proportion of patients receiving NACT+ACT with resection (17.3% vs. 23.9% vs. 19.7%).

Despite higher costs for treatments with curative intent compared to palliative treatments, patients receiving best supportive care had the highest costs per QALY gained. Most palliative patients live short in a poorer condition leading to relatively high costs. As the proportion of patients treated with best supportive care is highest in low probability hospitals this leads to higher costs per QALY. For both the societal and healthcare perspective, treating EC patients in medium instead of low probability hospitals and treating GC patients in high instead of medium probability hospitals is most cost-effective. Nonetheless, as the highest QALY is found for patients diagnosed in high probability hospitals and ICERs are far below €80,000/QALY, treating patients more frequently with curative intent, as is seen in high probability hospitals, could be recommended. Low and medium probability hospitals could attempt to adjust their treatment strategies comparable to high probability hospitals, however, much remains unknown about how high probability hospitals differ from the others.

After careful consideration, it was decided to exclude indirect medical costs made in gained life years, due to the poor prognosis of patients with EC and GC. As cancer is the cause of death in almost 90% of these patients,^40^ we expect that medical costs for other diseases in gained life years do not contribute substantially to total costs. Moreover, we did not discount costs and benefits. Costs were calculated for the patient’s whole trajectory instead of per year. For most patients, the primary treatment was performed in the first year after diagnosis, and possible secondary treatment after progression or recurrence in the second or third year after diagnosis. Therefore, since costs occur in similar years for most patients, we do not expect major relative cost differences per year between treatment strategies. Furthermore, ICERs would still be below €80,000/QALY when discounting costs and benefits.

Our study has several limitations. By using the top-down gross costing approach, total costs were estimated from resource use and costs of the average EC and GC patient. This approach tends to underestimate costs^41^; however, using patient-specific data on resource use and costs was beyond the scope of this analysis. Furthermore, costs of formal and informal care, and emergency department visits could not be included in our analysis, because these data were not available. Costs of GC resection were estimated in consultation with a medical specialist due to unavailability of GC surgery costs. Resource use and costs for palliative care (e.g. hospital admission days, diagnostics, medication) were difficult to estimate due to lack of data, and costs could thus be under- or overestimated. Moreover, for patients undergoing endoscopic resection or treatment without curative intent, mean utility values and costs of productivity loss were based on low numbers of questionnaires or could not be estimated at all. The low number of questionnaires did also not allow us to use separate utilities for patients with or without progression or recurrence, although it is likely that patients with progression or recurrence have a lower quality of life than other patients. Taking these limitations into consideration, our total costs are probably somewhat underestimated. It is however expected that even with higher medical costs, treating patients in hospitals with a high probability of treatment with curative intent remains cost-effective, and the ratio between the hospital tertiles would only change to a limited extent. The results of our sensitivity analyses support this. In terms of generalizability, the results may not apply to other countries with different healthcare systems, as the Dutch system lacks private EC and GC care, and ensures equal access regardless of income or insurance.

Currently, adjuvant treatment with nivolumab is becoming the standard of care for patients with EC that have residual disease in the specimen after undergoing nCRT and resection, contributing substantially to treatment costs and improving disease-free survival.^42^ This adjustment was not yet included in our analyses. This may influence comparisons between hospital categories, as although the proportion of patients receiving nCRT with resection were comparable, providing additional adjuvant treatment with nivolumab may not be similar between hospital categories in the future.

In conclusion, although variation in providing treatment with curative intent between hospitals affects health economics outcomes only to a limited extent, the differences in health outcomes at population level might still be meaningful. The gain in QALYs were slightly higher for patients diagnosed in high probability hospitals compared to low and medium probability hospitals, while associated costs were comparable. These findings suggest that costs should not be a limiting factor to treat patients with curative intent, for those patients who wish to.

## Supplementary Material

Cost_effectiveness_manuscript_28052025_supplementary_material_doaf057

## Notes

Specific author contributions: Data curation, Formal analysis, Methodology, Visualization, Writing—original draft: Saskia PM Truijen; Conceptualization, Formal analysis, Funding acquisition, Methodology, Project administration, Supervision, Writing—original draft, Writing—review & editing: Pauline AJ Vissers; Methodology, Writing—review & editing: Grard Nieuwenhuijzen; Writing—review & editing: Maurice Van der Sangen; Writing—review & editing: Peter Siersema; Writing—review & editing: Marije Slingerland; Writing—review & editing: Nadia Haj Mohammad; Writing—review & editing: Laurens V Beerepoot; Writing—review & editing: Mark van Berge Henegouwen; Writing—review & editing: Pieter Van der Sluis; Writing—review & editing: Camiel Rosman; Writing—review & editing: Ewout A Kouwenhoven; Writing—review & editing: Hüseyin Aktas; Writing—review & editing: Hanneke WM van Laarhoven; Methodology, Writing—review & editing: Carin Uyl-de Groot; Conceptualization, Formal analysis, Funding acquisition, Methodology, Project administration, Supervision, Writing—original draft, Writing—review & editing: Rob HA Verhoeven.

Financial support: This study was financially supported by the KWF Dutch Cancer Society (project number: 10895). The sponsor(s) of the open access fee are Maastricht University/Maastricht University Medical Center+ (UM/MUMC+).

Conflicts of interest: Rob H.A. Verhoeven received a research grant from Bristol Myers Squibb and a consultancy fee by Daiichi Sankyo, all paid to institution. Peter D. Siersema received research support from Pentax, The Enose Company, MicroTech, Lucid Diagnostics and Motus GI. Grard A.P. Nieuwenhuijzen received research support from Proctorship payments from Medtronic, and a research grant from CZ Assurance Company. Mark I. van Berge Henegouwen is consultant for Mylan, Johnson & Johnson, Alesi Surgical, B. Braun and Medtronic, and received unrestricted research grants from Stryker. All fees paid to institution. Marije Slingerland has an advisory role at Bristol-Myers Squibb and Lilly. Camiel Rosman received research grants from Johnson & Johnson and Medtronic. Ewout A. Kouwenhoven received research support from Proctor for Intuitive Surgical. Authors Saskia P.M. Truijen, Pauline A.J. Vissers, Maurice J.C. van der Sangen, Nadia H. Mohammad, Laurens V. Beerepoot, Pieter C. van der Sluis, Hüseyin Aktas, Hanneke W.M. van Laarhoven and Carin A. Uyl-de Groot declare no conflicts of interest.
