# Supplementary material for: A cost-effectiveness analysis of the effect of hospital variation in the probability of providing treatment with curative intent in potentially curable esophageal and gastric cancer patients
Source: Dis Esophagus. 2025 Jul 18;38(4):doaf057. doi: 10.1093/dote/doaf057 (PMC12272846; doi:10.1093/dote/doaf057)
Supplement: Cost_effectiveness_manuscript_28052025_supplementary_material_doaf057 [file cost_effectiveness_manuscript_28052025_supplementary_material_doaf057.docx]

**Supplementary Material**

| Supplementary Table 1. Unit costs (2020 €) and references for prices and units/proportions | | | |
| --- | --- | --- | --- |
| Resource | Cost per unit (2020 €) | Price reference | Unit/proportion reference |
| Direct medical costs | | | |
| *Consultations* |  |  |  |
| Consult (dietician) | 32 | [31] | Consultation specialist |
| Consult (physiotherapist) | 43 | [31] | Consultation specialist |
| Consult (all other specialisms) | 98 | [9] | NCR/Consultation specialist |
| Consult (during palliative care phase) | 98 | [9] | [24] |
| MDO (5 min x seven specialists) | 50 | [9] | Consultation specialist |
|  |  |  |  |
| *Diagnostics* |  |  |  |
| CT scan | 191 | [28] | NCR/Consultation specialist |
| PET-CT scan | 1124 | [28] | NCR/Consultation specialist |
| Esophagogastroscopy (incl. biopt) | 266 | [28] | NCR/Consultation specialist |
| Endoscopic ultrasound (EUS) esophagus | 855 | [28] | NCR/Consultation specialist |
| Echo neck and puncture | 85 | [28] | NCR/Consultation specialist |
| Diagnostic laparoscopy | 973 | [32] | NCR/Consultation specialist |
| Laboratory test | 12 | [28] | Consultation specialist |
| Tumor pathology | 128 | [28] | NCR |
| Endobronchial ultrasound (EBUS) | 774 | [28] | NCR/Consultation specialist |
| Electrocardiogram (ECG) | 50 | [28] | Consultation specialist |
|  |  |  |  |
| *Patient days* |  |  |  |
| ICU in-patient day | 2178 | [9] | NCR |
| In-patient day | 515 | [9] | NCR |
| Day-care visit oncology | 279 | [33] | Consultation specialist |
|  |  |  |  |
| *Resection* |  |  |  |
| Pre-operative screening (POS) | 98 | [9] | Consultation specialist |
| Endoscopic resection (EMR/ESD) | 2655 | [29] | NCR |
| Resection esophageal carcinoma |  |  |  |
| Resection (surgery, laboratory measurements, interventions, diagnostics, blood products, outpatient clinic, other costs) | 15495 | [34] | NCR |
| Hospital stay (ICU and general ward in-patient days) ^a^ | (1) 17030 (2) 15947 | [9] | NCR |
| Resection gastric carcinoma |  |  |  |
| Resection (surgery, laboratory measurements, interventions, diagnostics, blood products, outpatient clinic, other costs) | 8553 ^b^ | [29,34] | NCR |
| Hospital stay (ICU and general ward in-patient days) ^a^ | (1) 5085  (2) 11097 (3) 9917 | [9] | NCR |
|  |  |  |  |
| *Systemic therapies* (incl. medication, daycare visit(s) oncology, laboratory measurements, opt. peripherally inserted central venous catheter (PICC), opt. port-a-cath (PAC)) ^c^ *(no. of cycles x no. of weeks)* |  |  |  |
| Capecitabine/Cisplatin (5x1) | 1695 | [33,35,43] | NCR |
| Carboplatin/Paclitaxel (5x1) | 3100 | [33,35,44] | NCR |
| CAPOX (3x3) | 2850 | [33,35,45] | NCR |
| ECC (3x3) | 1905 | [33,35,43] | NCR |
| ECF (3x3) | 1611 | [33,35,46] | NCR |
| EOX/EOC (3x3) | 3240 | [33,35,43] | NCR |
| FLOT (4x2) | Neoadj: 5901 Adj: 6049 | [29,33,35,47,48] | NCR |
| FOLFOX (4x2) | 4524 | [33,35,49] | NCR |
| Other | Neoadj: 3304 Adj: 3125 | - | NCR |
|  |  |  |  |
| *Systemic/targeted therapies provided after recurrence or progression* ^d^  *(mean no. of cycles x no. of weeks)* |  |  |  |
| Capecitabine  (GC: 6x3) | 1260 | [33,35,50] | NCR |
| Capecitabine/Cisplatin/Trastuzumab  (EC: 5.5x3) | 9111 | [33,35,51] | NCR |
| CAPOX  (EC: 3.9x3, GC: 3.2x3) | EC: 3705 GC: 3040 | [33,35,45] | NCR |
| CAPOX/Trastuzumab (EC: 3.9x3, GC: 4.7x3) | EC: 8293  GC: 9920 | [33,35,52] | NCR |
| Carboplatin/Paclitaxel  (EC: 6.2x1, GC: 5.3x1) | EC: 3720 GC: 3286 | [33,35,44] | NCR |
| EOX (EC: 4x3) | 4320 | [33,35,43] | NCR |
| FOLFOX  (EC: 4.3x2, GC: 5.6x2) | EC: 5930 GC: 7401 | [29,33,35,49] | NCR |
| Paclitaxel/Ramucirumab (EC: 7.3x4, GC: 4.5x4) | EC: 63065 GC: 38876 | [33,35,53] | NCR |
| Other (second-line systemic therapy) | EC: 4347 GC: 3985 | - | NCR |
| Other (second-line targeted therapy) | EC: 23048 GC: 9920 | - | NCR |
|  |  |  |  |
| *Radiotherapy* ^e^ |  |  |  |
| Preparation | 1594 | [30] | Consultation specialist |
| External radiation | 426 | [29] | NCR |
|  |  |  |  |
| *Follow-up (€/month)* |  |  |  |
| Treatments with curative intent (incl. consultations, laboratory measurement) | 17 | [9] | Richtlijnendatabase.nl |
| Endoscopic resection (incl. consultations, laboratory measurement, endoscopies) | 58 | [9,28] | Richtlijnendatabase.nl/  Consultation specialist |
| Treatments without curative intent | 0 | - | Richtlijnendatabase.nl |
|  |  |  |  |
| *Palliative phase (€/month)* |  |  |  |
| Last three months (incl. consultations, medication, diagnostics, hospital stay) | 3262 | [9,25] | NCR and [24-26] |
| Before last three months (incl. consultations, medication, diagnostics) | 844 | [9,25] | NCR and [24,25] |
|  |  |  |  |
| *Treatment after recurrence or progression / best supportive care* |  |  |  |
| Systemic / targeted therapy (see above) |  |  |  |
| Radiotherapy | 5215 | [29,30] | NCR |
| Chemoradiation | 16622 | [29,30] | NCR |
| Resection (organ) | EC: 33532 GC: 13944 |  | NCR |
| Resection (local) | 2961 | [9,29] | NCR |
| Resection (metastasis) | 12068 | [9,36] | NCR |
| Endoprosthesis/stent | 2079 | [9,28] | NCR |
|  |  |  |  |
| Travelling expenses ^f^ | | | |
| Car | 3.00 + 0.19/km | [9] | [54] |
| Taxi | 2.95 + 2.66/km | [9] | [54] |
| Public transport | 0.20/km | [9] | [54] |
|  |  |  |  |
| Productivity loss costs | | | |
| Productivity loss costs per hour | 37.56 | [9] | POCOP |
| ^a^ Costs of hospital stay after surgery were calculated for each treatment category involving resection, by multiplying the cost of an ICU or general ward in-patient day by the mean days of ICU and general ward stay in the treatment category.  ^b^ The cost of gastric carcinoma surgery was absent in literature, therefore costs were estimated by using the ratio of ‘gastric carcinoma surgery : esophageal cancer surgery (€16,117 : €29,182)’ of the Amsterdam UMC Pricelist ^[29]^ with the price of esophageal cancer surgery. ^[34]^  ^c^ Standard numbers of cycles and doses were used. For every cycle involving IV-therapy a day-care visit at the oncology department and laboratory measurement were counted. Costs of applying a PICC or PAC system were taken into account if applicable.  ^d^ The mean number of cycles in the NCR data was used.  ^e^ The mean amount of fractions in the NCR data was used.  ^f^ Travelling expenses were calculated by multiplying return trip cost prices by the number of return trips to hospital per treatment category, assuming that 90% of patients traveled by car, 5% by taxi, and 5% by public transport, with 20% of hospital appointments combined. Median travel distance was 28 km for EC and 26 km for GC.^[54]^ | | | |

| Supplementary Table 2. Mean productivity loss costs and proportion of patients performing paid work per period and per treatment category for EC and GC ^a^ | | | | | | | | | | | | |
| --- | --- | --- | --- | --- | --- | --- | --- | --- | --- | --- | --- | --- |
| Treatment | Mean costs of productivity loss (SD) (€) | | | | | Proportion of patients performing paid work | | | | | ≥67 years | Mean costs (€) |
| Time periods | P1 | P2 | P3 | P4 | P5 | P1 | P2 | P3 | P4 | P5 |  |  |
| Esophageal cancer | | | | | | | | | | | | |
| nCRT and resection | 4617 (3551) | 11502 (7111) | 4799 (7404) | 2530 (5152) | 17064 (33459) | 33.2% | 32.1% | 29.1% | 29.4% | 21.2% | 47.7% | €10,983 |
| Resection, other | 6206 (5392) | 13131 (5485) | 4122 (6128) | 5382 (6350) | 10611 (12526) ^b^ | 33.3% | 35.0% | 34.1% | 35.1% | 25.0% | 49.9% | €12,610 |
| Endoscopic resection | - | - | - | - | - | - | - | - | - | - | 63.3% | - |
| Definitive chemoradiation | 6227 (3795) | 4068 (2186) ^b^ | 15708 (8917) | 2521 (5127) **^c^** | 8741 (14074) ^c^ | 18.6% | 14.7% | 13.1% | 7.0% | 7.4% | 72.7% | €4,637 |
| nCRT without resection | 4760 (3619) | 4695 (4280) | 8175 (7072) | 2521 (5127) | 8741 (14074) | 37.2% | 26.0% | 21.8% | 24.4% | 21.9% | 58.5% | €7,303 |
| Radiotherapy only | 1871 (2514) ^b^ | - | - | - | - | 13.3% | 0.0% ^b^ | 6.3% | 0.0% ^b^ | 0.0% ^d^ | 90.2% | €249 |
| Systemic therapy only | 3477 (4179) ^b^ | - | - | - | - | 25.0% | 25.0% ^b^ | 20.0% | 20.0% ^b^ | 0.0% ^d^ | 56.3% | €869 |
| Best supportive care ^d^ | - | - | - | - | - | - | - | - | - | - | 87.1% | €0 |
| Gastric cancer | | | | | | | | | | | | |
| NACT+ACT with resection | 3960 (4188) | 12761 (7956) | 2238 (2918) ^b^ | 5563 (5851) | 2704 (4684) ^b^ | 40.0% | 40.0% | 28.2% | 37.9% | 29.4% | 44.8% | €10,223 |
| NACT with resection | 5656 (4778) | 9980 (6431) ^b^ | 9873 (6212) | 223 (591) ^b^ | 0 (0) ^b^ | 23.5% | 17.2% | 17.5% | 15.9% | 11.1% | 65.7% | €4,809 |
| Resection, other | - | - | - | - | - | 0.0% | 14.3% ^b^ | 0.0% | 0.0% | 0.0% ^d^ | 82.1% | - |
| Endoscopic resection | - | - | - | - | - | - | - | - | - | - | 80.4% | - |
| Systemic therapy only | 7684 (4917) ^b^ | 4023 (5689) ^b^ | 5570 (2322) ^b^ | - | - | 50.0% ^b^ | 40.0% ^b^ | 50.0% ^b^ | - | - | 62.5% | €4,041 |
| Best supportive care ^d^ | - | - | - | - | - | - | - | - | - | - | 95.0% | €0 |
| ^a^ ‘-‘ means no data were available at the time period.  ^b^ Data are based on <10 patients.  ^c^ Productivity loss costs of EC patients undergoing nCRT without resection were used.  ^d^ No data were available, it was assumed no productivity loss costs were made after considering age and survival probability  **nCRT** Neoadjuvant chemoradiation. **NACT** Neoadjuvant chemotherapy. **ACT** Adjuvant chemotherapy. | | | | | | | | | | | | |

| Supplementary Table 3. Mean utility values and life years of EC and GC patients per treatment category ^a^ | | | | | | | | | | | |
| --- | --- | --- | --- | --- | --- | --- | --- | --- | --- | --- | --- |
| Treatment | Mean utility (SD) | | | | | Mean no. of years in period (SD) | | | | | Mean QALY (SD) |
| Time periods | P1 | P2 | P3 | P4 | P5 | P1 | P2 | P3 | P4 | P5 |  |
| Esophageal cancer | | | | | | | | | | | |
| nCRT and resection | 0.861 (0.131) | 0.851 (0.143) | 0.824 (0.154) | 0.844 (0.157) | 0.840 (0.160) | 0.11 (0.04) | 0.32 (0.13) | 0.47 (0.10) | 0.47 (0.10) | 2.67 (0.00) | 2.87 (1.11) |
| Resection, other | 0.842 (0.150) | 0.831 (0.175) | 0.788 (0.200) | 0.827 (0.220) | 0.839 (0.150) | 0.14 (0.09) | 0.32 (0.22) | 0.47 (0.11) | 0.47 (0.10) | 2.73 (0.00) | 2.83 (1.22) |
| Endoscopic resection | 0.944 (0.080) ^b^ | 0.777 (0.200) ^b^ | 0.841 (0.145) ^b^ | 0.723 (0.052) ^b^ | 0.789 (0.174) ^b^ | 0.15 (0.14) | 0.11 (0.25) | 0.49 (0.05) | 0.50 (0.03) | 3.42 (0.00) | 3.25 (1.20) |
| Definitive chemoradiation | 0.815 (0.162) | 0.838 (0.125) | 0.803 (0.185) | 0.819 (0.168) | 0.807 (0.201) | 0.13 (0.06) | 0.12 (0.10) | 0.46 (0.11) | 0.45 (0.12) | 2.00 (0.00) | 1.98 (0.96) |
| nCRT without resection | 0.858 (0.119) | 0.852 (0.157) | 0.854 (0.158) | 0.818 (0.195) | 0.853 (0.134) | 0.12 (0.06) | 0.10 (0.10) | 0.45 (0.12) | 0.43 (0.14) | 1.60 (0.00) | 1.61 (0.90) |
| Radiotherapy only | 0.662 (0.280) | 0.749 (0.168) ^b^ | 0.657 (0.332) | 0.745 (0.314) ^b^ | 0.697 (0.287) ^b^ | 0.11 (0.08) | 0.07 (0.11) | 0.38 (0.16) | 0.37 (0.17) | 1.06 (0.00) | 0.76 (0.57) |
| Systemic therapy only | 0.812 (0.107) | 0.706 (0.236) ^b^ | 0.750 (0.149) | 0.721 (0.124) ^b^ | 0.803 (0.059) ^b^ | 0.13 (0.06) | 0.18 (0.20) | 0.35 (0.19) | 0.35 (0.18) | 0.67 (0.00) | 0.75 (0.52) |
| Best supportive care ^c^ | - | - | - | - | 0.663 (0.324) ^b^ | 0.43 (0.54) | | | | | 0.29 (0.36) |
| Gastric cancer | | | | | | | | | | | |
| NACT+ACT with resection | 0.904 (0.120) | 0.840 (0.150) | 0.848 (0.145) | 0.856 (0.158) | 0.838 (0.189) | 0.12 (0.06) | 0.50 (0.10) | 0.48 (0.07) | 0.48 (0.08) | 2.82 (0.00) | 3.31 (1.03) |
| NACT with resection | 0.861 (0.148) | 0.812 (0.173) | 0.817 (0.187) | 0.866 (0.139) | 0.855 (0.157) | 0.12 (0.05) | 0.29 (0.13) | 0.45 (0.13) | 0.47 (0.10) | 2.56 (0.00) | 2.66 (1.20) |
| Resection, other | 0.837 (0.197) | 0.845 (0.163) | 0.838 (0.176) | 0.847 (0.141) | 0.808 (0.175) | 0.14 (0.09) | 0.06 (0.12) | 0.44 (0.14) | 0.47 (0.10) | 2.54 (0.00) | 2.27 (1.24) |
| Endoscopic resection ^d^ | 0.944 (0.080) ^b^ | 0.777 (0.200) ^b^ | 0.841 (0.145) ^b^ | 0.723 (0.052) ^b^ | 0.789 (0.174) ^b^ | 0.15 (0.18) | 0.01 (0.05) | 0.49 (0.07) | 0.49 (0.03) | 3.25 (0.00) | 3.07 (1.10) |
| Systemic therapy only ^e^ | 0.833 (0.067) ^b^ | 0.819 (0.151) ^b^ | 0.699 (0.214) ^b^ | 0.699 (0.214) ^b^ | 0.833 (0.067) ^b^ | 0.11 (0.07) | 0.16 (0.16) | 0.30 (0.19) | 0.35 (0.18) | 0.69 (0.00) | 0.62 (0.48) |
| Best supportive care ^c, d^ | - | - | - | - | 0.663 (0.324) ^b^ | 0.56 (0.67) | | | | | 0.38 (0.45) |
| ^a^ The number of life years in period 5 were calculated as the mean time in period 5 of patients diagnosed in 2015-2016.  ^b^ Mean utility is based on <10 patients.  ^c^ For patients receiving best supportive care the mean QALY was calculated as the mean utility of period 5 times the no. of years survival.  ^d^ Mean utilities of EC patients were used.  ^e^ Mean utilities of period 1 and 3 were used again for period 4 and 5. **nCRT** Neoadjuvant chemoradiation. **NACT** Neoadjuvant chemotherapy. **ACT** Adjuvant chemotherapy. | | | | | | | | | | | |

| Supplementary Table 4. Sensitivity analyses: ICERs from a societal perspective per hospital adjusted probability of treatment with curative intent ^a^ | | | |
| --- | --- | --- | --- |
| Parameters | Hospital adjusted probability of treatment with curative intent | | |
|  | Medium (2) vs. Low (1) | High (3) vs. Low (1) | High (3) vs. Medium (2) |
| Esophageal cancer |  |  |  |
| ICER | | | |
| Resection price -15% | -€3,900 | €2,150 | €4,167 |
| Resection price +15% | -€6,000 | €2,725 | €5,633 |
| Hospital day price -15% | -€3,833 | €2,267 | €4,300 |
| Hospital day price +15% | -€6,033 | €2,875 | €5,844 |
| Palliative phase €/month -15% | -€4,000 | €3,125 | €5,500 |
| Palliative phase €/month +15% | -€5,833 | €1,783 | €4,322 |
| QALY -15% | -€5,804 | €2,873 | €5,765 |
| QALY +15% | -€4,290 | €2,123 | €4,261 |
|  | Medium (2) vs. Low (1) | High (3) vs. Low (1) | High (3) vs. Medium (2) |
| Gastric cancer | | | |
| ICER | | | |
| Resection price -15% | €9,778 | €5,141 | -€7,233 |
| Resection price +15% | €11,096 | €6,506 | -€5,944 |
| Hospital day price -15% | €10,048 | €5,103 | -€7,533 |
| Hospital day price +15% | €11,030 | €6,341 | -€5,644 |
| Palliative phase €/month -15% | €10,987 | €6,331 | -€5,567 |
| Palliative phase €/month +15% | €10,083 | €5,109 | -€7,600 |
| QALY -15% | €12,399 | €6,728 | -€7,765 |
| QALY +15% | €9,164 | €4,973 | -€5,739 |
| ^a^ Four parameters were chosen to recalculate ICERs in case of -15% or +15% cost price: Resection price, hospital day price, palliative phase (€/month) price, and QALY. | | | |
